# Location and Dynamics of the Immunodominant CD8 T Cell Response to SIVΔnef Immunization and SIVmac251 Vaginal Challenge

**DOI:** 10.1371/journal.pone.0081623

**Published:** 2013-12-09

**Authors:** Arun K. Sasikala-Appukuttan, Hyeon O. Kim, Nikilyn J. Kinzel, Jung Joo Hong, Anthony J. Smith, Reece Wagstaff, Cavan Reilly, Michael Piatak, Jeffrey D. Lifson, R. Keith Reeves, R. Paul Johnson, Ashley T. Haase, Pamela J. Skinner

**Affiliations:** 1 University of Minnesota, Veterinary and Biomedical Sciences Department, Saint Paul, Minnesota, United States of America; 2 University of Minnesota, Microbiology Department, Minneapolis, Minnesota, United States of America; 3 University of Minnesota, School of Public Health, Division of Biostatistics, Minneapolis, Minnesota, United States of America; 4 AIDS and Cancer Virus Program, Leidos Biomedical Research, Inc., (formerly Science Applications International Corporation–Frederick, Inc.), Frederick National Laboratory, Frederick, Maryland, United States of America; 5 Division of Immunology, New England Primate Research Center, Harvard Medical School, Southborough, Massachusetts, United States of America; University of Hawaii Manoa, United States of America

## Abstract

Live-attenuated SIV vaccines (LAVs) have been the most effective to date in preventing or partially controlling infection by wild-type SIV in non-human primate models of HIV-1 transmission to women acting by mechanisms of protection that are not well understood. To gain insights into mechanisms of protection by LAVs that could aid development of effective vaccines to prevent HIV-1 transmission to women, we used in situ tetramer staining to determine whether increased densities or changes in the local distribution of SIV-specific CD8 T cells correlated with the maturation of SIVΔnef vaccine-induced protection prior to and after intra-vaginal challenge with wild-type SIVmac251. We evaluated the immunodominant Mamu-A1*001:01/Gag (CM9) and Mamu-A1*001:01/Tat (SL8) epitope response in genital and lymphoid tissues, and found that tetramer+ cells were present at all time points examined. In the cervical vaginal tissues, most tetramer+ cells were distributed diffusely throughout the lamina propria or co-localized with other CD8 T cells within lymphoid aggregates. The distribution and densities of the tetramer+ cells at the portal of entry did not correlate with the maturation of protection or change after challenge. Given these findings, we discuss the possibility that changes in other aspects of the immune system, including the quality of the resident population of virus-specific effector CD8 T cells could contribute to maturation of protection, as well as the potential for vaccine strategies that further increase the size and quality of this effector population to prevent HIV-1 transmission.

## Introduction

While there have been heartening advances in antiretroviral therapies that have converted HIV-1 infection in the developed world to a chronic but manageable disease, it is clear that better measures for prevention are needed to contain the continuing growth of a pandemic that has already claimed more than 25 million lives and currently afflicts 34 million people [1]. This is especially the case for women because of the increasing feminization of the pandemic epicenter in sub-Saharan Africa where 75 percent of the HIV-1 infected population ages 15–24 are females [1], [2]. Development of a prophylactic vaccine to prevent HIV infection represents the most effective, economical, and universal solution to achieving this goal, but thus far in human trials, vaccine candidates have been at best marginally effective [3], [4]. The SIV-infected rhesus macaque model of HIV is a powerful system being used to gain insights into HIV/SIV pathogenesis and aid in the development of an effective HIV vaccine. Some promising vaccine strategies tested in rhesus macaques include vaccines that mediate sustained presentation of SIV viral epitopes, such as live-attenuated SIVs (LAV) [5] and vaccines based on persistent recombinant herpes virus vectors, including recombinant cytomegalovirus (CMV) vaccines [6], [7] and recombinant rhadinovirus vaccines [8]. Arguably the most effective vaccine evaluated to date in this system is the LAV SIVΔnef which suppressed viral replication in 95% of macaques challenged with wild-type (WT)-SIV, with 50% of challenged animals showing apparent sterilizing immunity[5], [9]–[15], and delayed the acquisition of WT-SIV infection after repeated low dose challenge [16]. Recent macaque studies using other vaccine approaches have shown promise, including vaccination with recombinant yellow fever virus and recombinant adenovirus vectors expressing immunodominant CD8 T cell epitopes, which demonstrated that virus-specific CD8 T cells can control WT-SIV infection [17]. Many other strategies including using viral DNA, viral particles and/or recombinant non-persisting viral vectors that express viral epitopes have also shown promise in suppressing viral replication and/or preventing infection from WT-SIV (a few of which we cite here) [18]–[21].

While there has been much recent progress in employing nonhuman primate models to aid development of an effective HIV vaccine, using a variety of different vaccine approaches, the efficacy of the LAV SIVΔnef remains a benchmark for the field, despite the fact that the underlying mechanism(s) of vaccine protection remain to be elucidated. Thus, the robust protection afforded by intravenous (iv) SIVΔnef vaccination against WT-SIV challenge by iv, rectal and vaginal routes [5], [9]–[16], supports studies of SIVΔnef vaccination to identify correlates of protection that could provide insight and principles to guide further development of an effective HIV-1 vaccine. To that end, we investigated and report here an analysis of the densities and locations after SIVΔnef vaccination of virus-specific CD8 T cells that recognize immunodominant epitopes in Gag and Tat in the genital and lymphoid tissues of rhesus macaques.

The hypothesis underlying these studies was that SIVΔnef vaccination might protect in part by converting the “too little and too late” CD8 T cell response to these immunodominant epitopes in unvaccinated animals [22], [23] to an “enough and soon enough” response in the vaccinated animals, sufficient to contain infection at the portal of entry or the lymphoid tissues (LTs) to which infection subsequently spreads. This hypothesis was founded in previous studies in the SIV-rhesus macaque vaginal challenge model [22], [23], which models HIV-1 transmission to women, where the CD8 T cell response was described as “too little and too late” for the following reasons. First, the peak of the immunodominant response occurred after the peak of virus replication in the lymphoid tissues (LT) that are the principal sites of virus production, persistence and pathology, such as the massive depletion of CD4 T cells in the gut, and thus too late to prevent this loss. Second, the magnitude of the CD8 T cell response was also insufficient to achieve the high effector to target cell ratios (E:T) in vivo in systemic LTs that correlate with 100-fold reductions in tissue viral load (VL) compared to peak VLs [24], and thus too little in that sense to control infection. However, in the cervical vaginal tissues, the higher E:T ratios were associated with reductions from peak of this magnitude [24] and thus the response is mainly too late. This latter finding suggested that a resident or rapid responder population of virus-specific CD8 T cells at the portal of entry and throughout the LTs to which infection spreads could be “enough and soon enough” to contain infection locally or after dissemination.

We therefore undertook studies of the location and dynamics of the immunodominant CD8 T cell response to vaccination with SIVmac239-Δnef and the response in vaccinated animals to high dose vaginal challenge with wild type SIVmac251 (hereafter, WT-SIV) to see if vaccination might induce and maintain a resident or rapid responder population of CD8 T cells that would be enough and soon enough to contain infection at the portal of entry or after dissemination to LTs. We focused on a comparison of samples obtained 5 and 20 weeks after SIVΔnef vaccination and after vaginal challenge at 20 weeks with WT-SIV. A comparison of 5 and 20 weeks after SIVΔnef vaccination is pertinent to this study because protection provided by live-attenuated SIVΔnef vaccination takes 15–20 weeks to “mature.” Animals challenged with WT-SIV before 15 weeks post-SIVΔnef vaccination show little to no suppression of viral replication, but when challenged after 15 weeks post-vaccination, 95% of the animals suppress viral replication with 50% showing apparent sterilizing immunity [12], [25]. This maturation process suggests involvement of adaptive immune components, including fully developed memory responses, in protection, and thus a CD8 T cell correlate of protection might be identified by comparing SIV-specific CD8 T cells in cervical vaginal tissues and LTs at 5 weeks post-SIVΔnef vaccination when the animals are not protected, with 20 weeks post-vaccination when there is sterilizing protection in half the animals and significant reductions in VLs in the remainder compared to unvaccinated controls.

## Materials and Methods

### Ethics statement

The 16 female Mamu-A1*001:01 (formerly Mamu A*01 [*26*]) Indian-derived rhesus macaque monkeys (Macacca mulatta) described in this study were housed at the New England Primate Center (NEPRC) in accordance with the regulations of the American Association of Accreditation of Laboratory Animal Care and the standards of the Association for Assessment and Accreditation of Laboratory Animal Care International. All protocols and procedures were approved by the relevant Institutional Animal Care and Use Committee which was the Harvard Medical Area (HMA) Standing Committee on Animals at Harvard Medical School. All animals were housed indoors in an SOP-driven, AAALAC-accredited facility. Husbandry and care met the guidance of the Animal Welfare Regulations, OLAW reporting and the standards set forth in The Guide for the Care and Use of Laboratory Animals. All research animals were enrolled in the NEPRC behavioral management program, including an IACUC-approved plan for Environmental Enrichment for research primates. This program included regular behavioral assessments, and provision of species appropriate manipulanda, and foraging opportunities. This protocol had an IACUC-approved exemption from social housing based on scientific justification. Primary enclosures consisted of stainless steel primate caging provided by a commercial vendor. Animal body weights and cage dimensions were regularly monitored. Overall dimensions of primary enclosures (floor area and height) met the specifications of The Guide for the Care and Use of Laboratory Animals, and the Animal Welfare Regulations (AWR's). Further, all primary enclosures were sanitized every 14 days at a minimum, in compliance with AWRs. Secondary enclosures (room level) met specifications of The Guide with respect to temperature, humidity, lighting and noise level. The animals were provided ad lib access to municipal source water, offered commercial monkey chow twice daily, and offered fresh produce a minimum of three times weekly. Light cycle was controlled at 12/12 hours daily. The animals were subject to twice daily documented observations by trained animal care and veterinary staff, and enrolled in the facility's environmental enrichment, and preventative health care programs. Euthanasia took place at defined experimental endpoints using protocols consistent with the American Veterinary Medical Association (AVMA) guidelines. Animals were first sedated with intramuscular ketamine hydrochloride at 20 mg/kg body followed by sodium pentobarbital (≥100 mg/kg) intravenously to achieve euthanasia.

### Vaccination and Vaginal challenge

Animals were negative for antibodies to HIV type 2, SIV type D retrovirus, and simian T-cell lymphotropic virus type 1. Animals were vaccinated by infecting intravenously with 25 ng of a stock of SIVmac239Δnef supplied by Dr. Ronald Desrosiers (NEPRC), and, when indicated, challenged vaginally twice on the same day (separated by 4 hours) with 10^5^ TCID_50_ of SIVmac251 supplied by Dr. Christopher Miller (CNPRC).

### Tissue collection and processing

At the time of euthanasia, genital tissues including, vagina (vestibule, middle, and near cervix), cervix (endo and ecto), uterus, and lymphoid tissues including, genital, mesenteric, axillary, and inguinal lymph nodes, spleen, and ileum were collected from each animal. Freshly dissected tissues were placed in tubes containing chilled RPMI media with heparin (100ug/ml or 18.7 U/ml) added as an RNase inhibitor and were shipped overnight on ice from the New England Primate Research Center to the University of Minnesota.

### In situ tetramer staining combined with immunohistochemistry

Animals with Mamu-A1*001:01 MHC class 1 alleles were selected for this study because, in this model system, immunodominant CD8 T cell responses to Gag and Tat epitopes represent approximately 70% of the total CD8 T cell response [27], and we have MHC tetramer reagents and methods available to stain the immunodominant SIV-specific CD8 T cells in situ. In situ tetramer staining combined with immunohistochemistry was performed similarly as previously described [28], [29]. Biotinylated Mamu-A1*001:01 molecules loaded with Gag181–189 CTPYDINQM (CM9) peptides were purchased from Beckman Coulter Immunomics. Mamu-A1*001:01 molecules loaded with Tat STPESANL (SL8) peptides or an irrelevant negative control peptide FLPSDYFPSV (FLP) from the hepatitis B virus core protein were obtained from the NIH Tetramer Core Facility (hereafter these molecules are referred to as Mamu-A1/Gag, Mamu-A1/Tat, and Mamu-A1/FLP). Tetramers were made by mixing 6 aliquots of FITC-labeled ExtraAvidin (Sigma) with MHC monomers over the course of 8 hours to a final molar ratio of 1:4.5. For tissue sectioning, fresh tissues were embedded in low melt agarose and cut into 200 micron thick sections. For the primary incubation, tissues where incubated with MHC tetramers at a concentration of 0.5 ug/ml in 1 ml of cold phosphate buffered saline containing 100 mg/ml heparin (PBS-H) with 2% normal goat serum at 4°C overnight. Sections were then washed with chilled PBS-H, fixed with 4% paraformaldehyde for 2 hours at room temperature, and again washed with PBS-H. For antibody counterstaining, prior to secondary incubation, tissues were boiled three times in 0.01 M Urea to expose epitopes, then permeabilized and blocked with PBS-H containing 0.3% triton X-100 and 2% normal goat serum for 1 hour. For the secondary incubation, sections were incubated with rabbit anti-FITC antibodies (BioDesign) diluted 1∶10,000 in blocking solution at 4°C on a rocking platform overnight. For the tertiary incubation, sections were washed with PBS-H and incubated with Cy3-conjugated goat anti-rabbit antibodies (Jackson ImmunoResearch) diluted 1∶5000 in blocking solution for 1 to 3 days, then washed again, post-fixed with 4% paraformaldehyde for 1 h, and mounted on slides with warmed glycerol gelatin (Sigma) containing 4 mg/ml *n*-propyl gallate. Sections were counterstained with several combinations of antibodies during primary, secondary, or tertiary incubations including: primary rat-anti-human CD8 antibodies diluted 1∶200 (AbD Serotec clone YTC182.20) or rat-anti-human CD3 antibodies diluted 1∶200 (AbD Serotec clone CD3-12) followed by tertiary Cy5-conjugated anti-rat antibodies (Jackson ImmunoResearch) diluted 1∶2000, and secondary mouse anti-CD20 antibodies diluted 1∶200 (Novacastra clone L26) or mouse anti-human Ki67 diluted 1∶200 (Vector clone MM1) followed by tertiary Alexa 488-conjugated goat anti-mouse antibodies (Molecular Probes) diluted 1∶2000 and goat anti-human IgM-Dylight 649 (1∶2000) (Jackson ImmunoResearch).

### Imaging

Confocal images were collected using a FluoView 1000 confocal microscope with a 20× objective and 3 µm z-steps. Confocal z-series were collected from approximately 5 µm from surface of the tissue section to as deep as the antibody counter-staining penetrated, approximately 35 µm into the tissue. Three-dimensional montage images of multiple 800×800 pixel 200X Z-scans were created using Olympus FluoView Viewer software.

### Quantification of cells

To determine the density of SIV-specific CD8 T cells in tissues, cells stained with Mamu-A1/Gag, Mamu-A1/Tat, and negative control Mamu-A1/FLP tetramers were counted in single z-scan montages of lymphoid and genital tissues. We stepped up and down through the z-scans to distinguish tops and bottoms of cells from non-specific background staining. Olympus FluoView Viewer software was used to delineate tissue areas and count tetramer^+^ cells in delineated areas. Image J software was then used to measure the areas delineated for cell counts. Because tetramer^+^ cells were most highly concentrated within the lamina propria of genital tissues, and because amounts of epithelium, lamina propria and underlying muscularis varied between samples and sections, in genital tissues we focused our quantification on tetramer^+^ cells in the lamina propria. Multiple confocal fields were evaluated for each sample covering 0.7 to 19 mm^2^, and averaging 3.1 mm^2^ in lymphoid tissue samples, and covering 0.23 to 14 mm^2^, averaging 2.2 mm^2^ in genital lamina propria samples. Data are presented as the total numbers of tetramer^+^ cells counted divided by the total area measured for each tissue from each animal. For the determination of the percentage of tetramer^+^ cells that were Ki67^+^, we quantified all of the whole tetramer^+^ cells present in each z-series montage image. We tested for differences between independent groups using a 2 sample t-test with common variance.

### SIVmac251 quantification

A real time qRT-PCR assay employing primers that specifically amplify WT-SIVmac251, even in the presence of SIVΔnef sequences, was used to quantify SIVmac251in plasma essentially as described [30].

### Association of viral load and SIV-specific CD8 T cell concentrations

To test for an association between plasma viral load and Mamu-A1/Gag and Mamu-A1/Tat-binding cell concentration simultaneously, a multivariate linear model was fit to the logarithms of all of the variables with the 2-vector of Mamu-A1/Gag and Mamu-A1/Tat values as the response variable and viral load as the predictor variable. These models allowed for correlation from Mamu-A1/Gag and Mamu-A1/Tat-binding cell levels within the same individual. Parameters were estimated using maximum likelihood and standard errors were obtained by numerically inverting the observed Fisher information matrix and taking the square root of the diagonal entries. Wald tests were used to test for associations between explanatory variables (Mamu-A1/Gag and Mamu-A1/Tat-binding cell levels) and viral load. This analysis was conducted separately for each tissue. Correlations were estimated using Pearson's correlation coefficient; however, the test for an association was based on the Wald test from the multivariate linear model. Such an approach uses the correlation between Mamu-A1/Gag and Mamu-A1/Tat-binding cells to get a test with improved power as it provides more precise estimates of the parameters.

## Results

### Experimental Design

**Figure 1:**
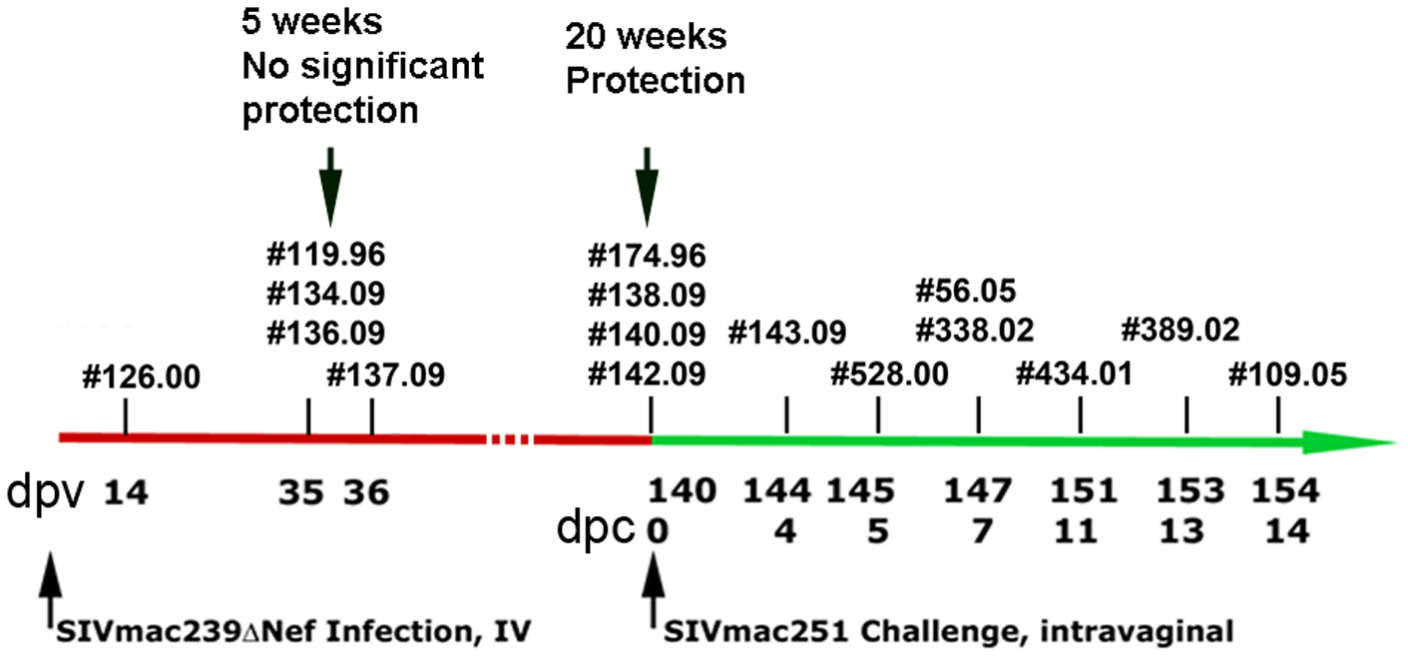
Study overview. Mamu-A1*001:01 rhesus macaques were vaccinated intravenously with SIVmac239Δnef and intravaginally challenged 20 weeks later with WT-SIV. Individual animal numbers are indicated at the day post-SIVΔnef vaccination (dpv, upper row) or day post-challenge with WT-SIV (dpc, lower row) that animals were evaluated.

The “enough and soon enough” hypothesis predicts: 1) that SIV-delta nef vaccination would elicit/establish a resident or rapid responder population of CD8 T cells located at the right time and place to contain infection following vaginal challenge at the portal of entry, or after dissemination to LTs; and 2) that the resident or responder populations will be distinguished by location, numbers or functions that might correlate with the maturation of protection between 5 and 20 weeks after SIVΔnef vaccination. We examined these predictions (see Figure 1 for a schematic overview) in 16 Mamu-A1*001:01 rhesus macaques vaccinated intravenously with SIVΔnef: after vaccination, but before protection is established, analyzing samples obtained at 2 weeks (n = 1 animal) and 5 weeks (n = 4 animals) after SIVΔnef vaccination and after protection has “matured” at 20 weeks (n = 4 animals). We also analyzed samples obtained in the first 2 weeks following high dose vaginal WT-SIV challenge at 20 weeks post-SIVΔnef vaccination (n = 7 animals), using in situ tetramer staining (IST) and complementary techniques to determine in genital and lymphoid tissues the location, numbers, and other characteristics of the CD8 T cells specific for immunodominant epitopes in Gag and Tat.

### Location of SIV-specific CD8 T cells in situ

We determined the location of immunodominant SIV epitope Gag- and Tat-specific CD8 T cells in cervix, vagina, draining lymph nodes, peripheral lymph nodes, spleen and ileum tissues, using in situ MHC-tetramer staining [28], [29] with Mamu-A1/Gag and Mamu-A1/Tat tetramers. As a negative control, tissues were stained with Mamu-A1 tetramers loaded with an irrelevant peptide (Mamu-A1/FLP). T cells and B cells were also visualized in the tissue sections by immunohistochemisty using antibodies directed against CD8, CD3, and CD20. We detected CD8^+^CD3^+^CD20^−^Mamu-A1/Gag and Mamu-A1/Tat tetramer^+^ T cells distributed amongst other CD8 T cells in the lymphoid and genital tissues from animals evaluated both before and after the predicted maturation of protection, and after challenge with WT-SIV.

**Figure 2:**
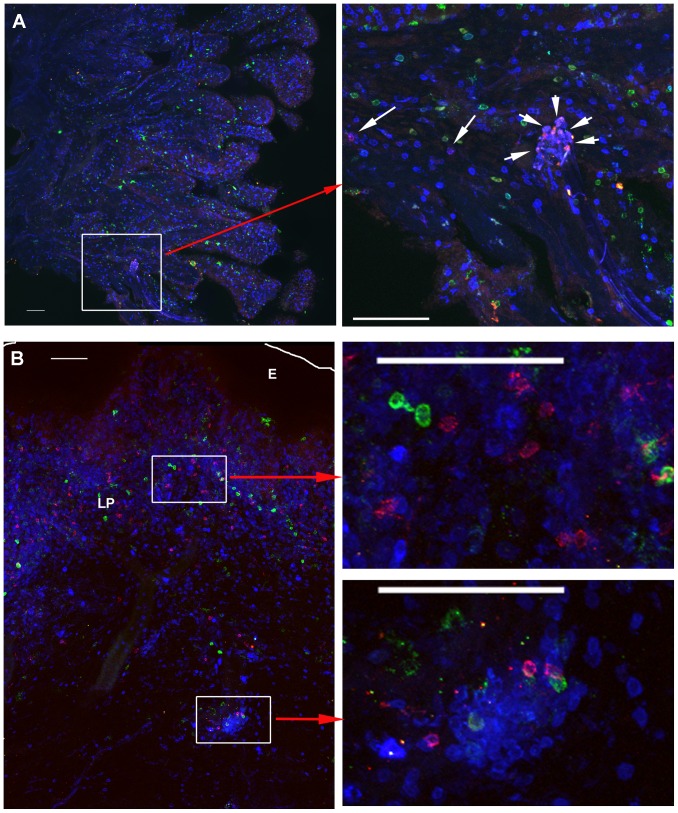
Localization of SIV-specific CD8^+^ T cells in cervix and vagina. A) Top panels show representative images of endocervix from an animal (137.09) at 5 weeks post-SIV-Δnef vaccination as examples of the low end of the spectrum of levels of tetramer+ detected in genital tissues amongst the whole cohort. Mamu-A1/Gag tetramer^+^ cells are red to pink, CD8^+^ T cells are blue, and CD20^+^ cells are green. B) In the bottom panels Mamu-A1/Tat MHC-tetramer-binding cells are red to pink, CD3^+^ T cells are blue, and CD20^+^ B cells are green from a representative image of vagina from animal #338.02, 21 weeks post-SIV-Δnef vaccination and 7 days following vaginal challenge with WT-SIV, as an example of the higher end of the spectrum of levels of tetramer^+^ cells detected in genital tissues amongst the whole cohort. The edge of the epithelium (E) is indicated with a white line; lamina propria indicated by LP. For both A) and B), the left panel shows a montage of several confocal projected z-series images (projected z-series are images collected a different depths stacked on top of each other), collected with a 20× objective. The right panels shows enlargements with several red to pink tetramer^+^ cells evident (indicated by arrows in A) localized diffusely as well as within small T cell aggregates. Scale bars = 100 µm.

At the portal of viral entry in cervical and vaginal tissues, tetramer^+^ cells were predominantly located within the lamina propria (Figure 2), but tetramer^+^ cells were also detected in the epithelial layer in approximately half of the animals at each time point. When detected in the genital epithelium, tetramer^+^ cells were in lower abundance relative to the lamina propria (3–25 fold less), and tended to be most concentrated near the border of the epithelium and lamina propria. The predominant localization of SIV-specific-tetramer^+^ cells in the lamina propria of genital tissues is similar to what we found in genital tissues from non-vaccinated WT-SIV infected rhesus macaques [31] and SHIV 89.6 vaccinated rhesus macaques (P.J. Skinner unpublished results), and this localization is consistent with the previously described localization of CD8 T cells in genital tissues from rhesus macaques [32] and women [33] indicating that the localization of SIV-specific CD8 T cells identified here is not specific for SIVΔnef vaccination.

**Figure 3:**
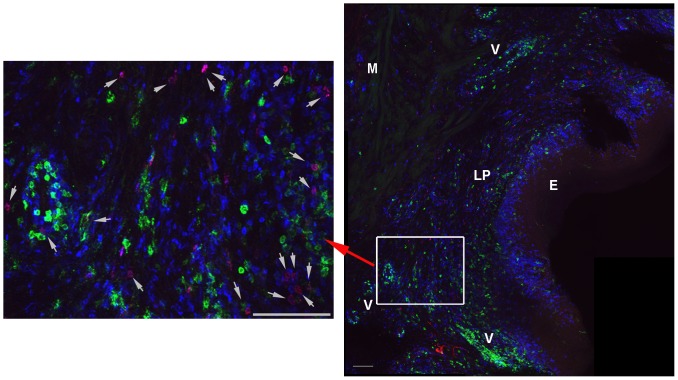
Localization of SIV-specific CD8 T cells in vagina at 14 days post-vaccination. Representative confocal image showing Mamu-A1/Gag tetramer^+^ cells (red), CD8 (blue), and CD20 (green). The right panel shows a montage of projected z-series of several 200× fields with the epithelium (E), lamina propria (LP), muscularis (M), and areas with vessels (V) indicated. In the enlarged image on the left, tetramer^+^ cells are indicated with arrows. Scale bars are 100 µm.

**Figure 4:**
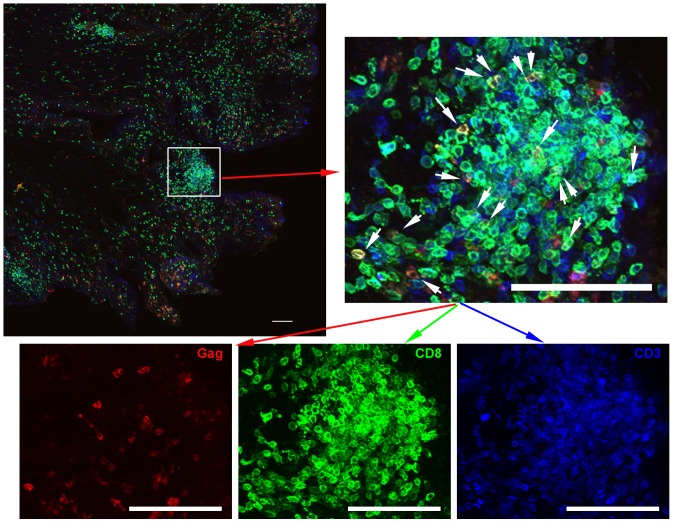
Most CD3^+^ T cells were CD8^+^ in genital lymphoid aggregates. Representative images of endocervix section stained with Mamu-A1/Gag tetramers (red), CD8 antibodies (green), and CD3 antibodies (blue) from animal #338.02. Tetramer+ cells are indicated with white arrows in the enlargement. The bottom panels show individual tetramer and antibody stains. Note most CD3^+^ T cells are CD8^+^. The top left panel shows a montage of projected z-series of several 200× fields. Scale bars = 100 µm.

**Figure 5:**
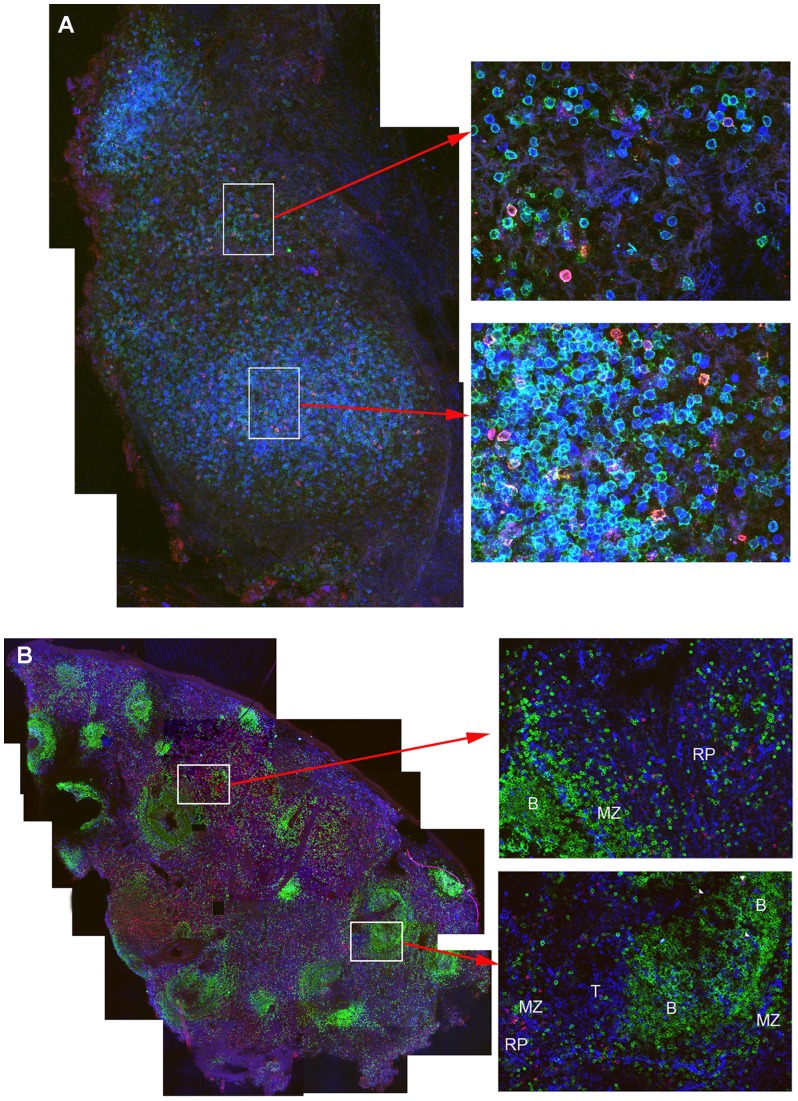
Localization of SIV-specific CD8^+^ T cells in lymph nodes and spleen. The representative images shown in are A) from an axillary lymph node from an animal (119.96) 5 weeks post-SIV-Δnef vaccination. Mamu-A1/Tat tetramer^+^ cells are red to pink, CD8^+^ T cells are green, and CD3^+^ T cells are blue. The left panel shows a montage of several fields containing projected z-scans collected with a confocal microscope 20× objective. Enlargements to the right show individual fields of projected z-scans collected with a 60× objective. The representative images shown in B) are from a spleen section from an animal (119.96) at 5 weeks post-SIVΔnef vaccination. Mamu-A1/Gag tetramer^+^ cells are red to pink colored, CD8^+^ T cells are blue, and CD20^+^ B cells are green. Tetramer^+^CD8^+^ cells localized with other CD8 T cells and were most abundant in the red pulp (RP) and marginal zones (MZ), with relatively fewer located in T cell zones (T) and in B cell follicles (B). The left panel shows a montage of several confocal microscope fields of projected Z-scans stitched together, collected with a 10× objective. Enlargements show individual confocal z-scans of single 200× fields.

In proximal, mid, and distal vagina tissues at 2 weeks post-vaccination, many T and B cells including tetramer^+^ cells surrounded vessels (Figure 3), some of which were clearly arterial. In addition, at all time points, tetramer+ cells were located within lymphoid aggregates in the cervix and vagina. These lymphoid aggregates, comprised of predominantly CD8 T cells (Figure 2, and Figure 4), were located throughout the lamina propria from just beneath the epithelium to near the muscularis (Figure 2), as well as within the muscularis, and ranged in size from ∼60 µm to over 500 µm in diameter. There were no obvious distinctions in the location of tetramer+ cells in genital tissues over the time course for development and maturation of LAV-SIVΔnef induced protection, between 5 and 20 weeks post-vaccination, or after challenge with WT-SIV. In lymph nodes, tetramer^+^ cells were primarily located in T cell zones (Figure 5A), whereas in spleen, tetramer^+^ cells were most abundant in the red pulp, with fewer cells located in the T cell zone and B cell follicles in the marginal zones (Figure 5B). In tissue sections of ileum, tetramer^+^ cells were located throughout the lamina propria and Peyer's patches (Figure S1). Within all of the lymphoid tissues, tetramer^+^ cells were often, but not always, in relatively lower abundance or largely absent from B cell follicles (Figure 5B). When there were tetramer^+^ cells in the follicles, they were often most abundant at the edge adjoining the T cell zone. No observed change in location of tetramer^+^ cells was observed within lymphoid tissues, between 5 and 20 weeks post-vaccination, or after challenge with WT-SIV.

### Abundance, activation and proliferative state of SIV-specific CD8 T cells in situ

**Figure 6:**
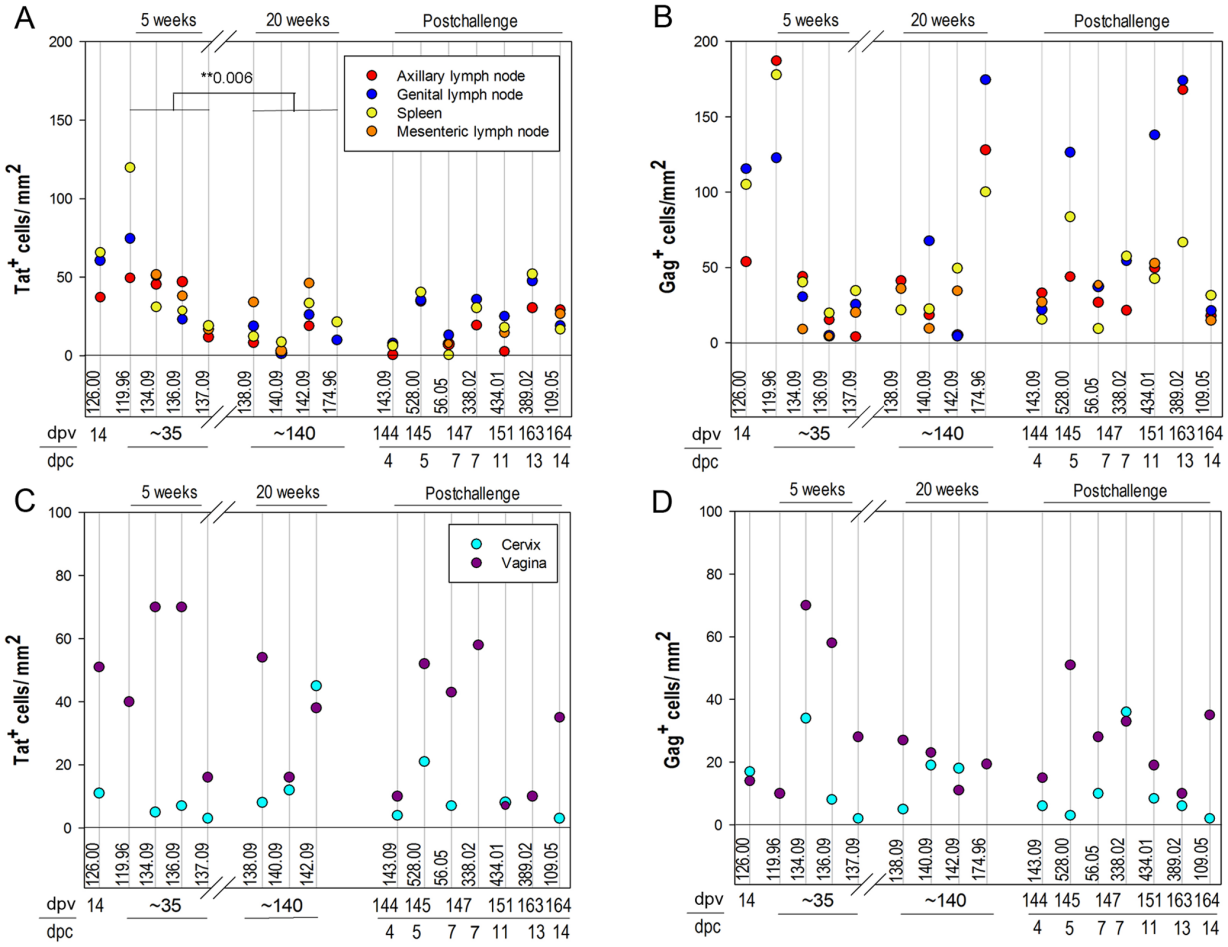
Abundance of SIV-specific CD8 T cells in lymphoid and genital tissues. The number of MHC-tetramer^+^ cells per mm^2^ in lymphoid tissues is presented in panels A) and B) and in the lamina propria of genital tissues in panels C) and D). The tissue density of Mamu-A1/Tat stained cells are shown in A) and C), and Mamu-A1/Gag stained cells shown in B) and D). Animal numbers, days post-SIVΔnef vaccination (dpv) and days post-challenge (dpc) are indicated below each graph. Negative control sections stained with Mamu-A1/FLP showed a range of 0-3.4 cells/mm^2^ and average of 1.25 cells/mm^2^ in lymphoid tissues, and 0 cells/mm^2^ in the genital tissues.

**Figure 7:**
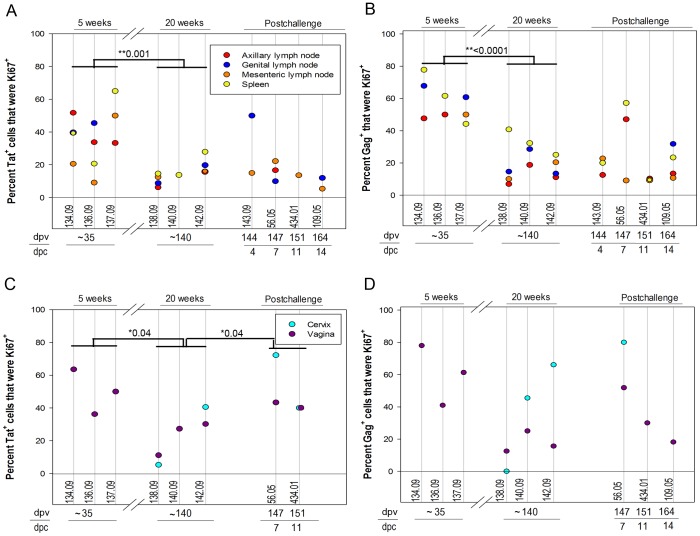
The percentage of tetramer^+^ SIV-specific CD8 T cells that were expressing the proliferation marker Ki67. The percentage of Mamu-A1/Tat tetramer^+^ cells (A and C) and Mamu-A1/Gag tetramer^+^ cells (B and D) that were Ki67^+^ in genital lymph nodes, axillary lymph nodes, mesenteric lymph nodes, and spleen is shown in the top panels and in vagina and cervix is shown in the bottom panels. Animal numbers, days post-SIVΔnef vaccination (dpv) and days post-challenge with WT-SIV (dpc) at necropsy are indicated below each graph. Significant decreases in the percentage of tetramer stained cells that were Ki67^+^ was observed in animals at 5 weeks (∼35 dpi) compared to 20 weeks (∼140 dpi) post-SIVΔnef vaccination. A significant increase in the percentage of Mamu-A1/Tat tetramer^+^ cells was observed in genital tissues during the first two weeks after challenge compared to animals just prior to challenge. *p-values are indicated. No significant difference was observed with Mamu-A1/Gag or Mamu-A1/Tat tetramer^+^ cells just prior to challenge compared to the first two weeks after challenge in lymphoid tissues or with MamuA1/Gag staining in genital tissues.

We determined the population densities and activation and proliferative state of tetramer^+^ SIV-specific CD8 T cells in situ in the lamina propria of cervix and vagina, as well as in draining lymph nodes, peripheral lymph nodes, and spleen, by IST staining, and staining for Ki67 as a marker for activation and proliferation (Figures 6 and 7). Tat-tetramer^+^ cells were less frequent in lymphoid tissues (Figure 6A), but not in cervical vaginal tissues (Figure 6C), at 20 weeks post-vaccination compared to 5 weeks post-vaccination, consistent with initial expansion and then contraction of the immune response associated with the declining SIVΔnef replication described elsewhere, as well as the evolution of escape in the Tat SL8 epitope (R. K. Reeves et al., ms. in preparation). Expression of the cell activation and proliferation marker Ki67 in SIV-specific CD8 T cells in situ at these sites was also consistent with this interpretation for both in LTs and cervical vaginal tissues (Figure 7). However, remarkably high percentages, averaging 38%, and ranging as high as nearly 80%, of tetramer^+^ cells were Ki67^+^ in the genital tissues (Figure 7C, D). SIVΔnef RNA was detected within lymphoid tissues of all animals at all time points (A.T. Haase and J.D. Lifson et al., manuscript in preparation), supporting the idea that ongoing SIVΔnef replication could be sustaining activation and ongoing proliferation of SIV-specific CD8 T cells in LTs and genital tissues. While the abundance of Mamu-A1/Tat-specific tetramer^+^ cells did not increase significantly following vaginal challenge, either in LTs or cervical vaginal tissues, there was a marginally significant increase in Mamu-A1/Tat-tetramer^+^ Ki67^+^ cells following challenge in cervical vaginal tissues, consistent with a recall response (Figure 7C).

The numbers of Mamu-A1/Gag-tetramer^+^ cells did not contract significantly between 5 and 20 weeks post-SIVΔnef vaccination (Figure 6B, D), but, in LTs, the frequency of Mamu-A1/Gag-tetramer^+^Ki67^+^ cells did decrease significantly (Figure 7B). However, there was no evidence for a recall response of Mamu-A1/Gag-tetramer^+^ cells from changes in abundance (Figure 6B, D) or increases in Ki67^+^ cells (Figure 7B, D) following vaginal challenge.

### Association of viral load and SIV-specific CD8 T cell levels in lymph nodes

**Figure 8:**
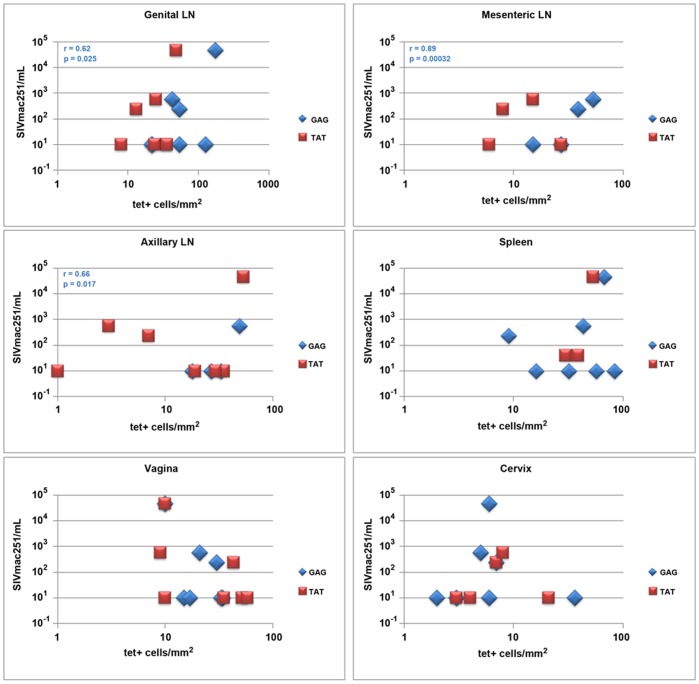
Comparison of plasma WT-SIV virus load and tissue densities of tetramer^+^ SIV-specific CD8 T cells. Tissue densities of Mamu-A1/Gag (blue) and MamuA1/Tat (red) tetramer^+^ cells for each tissue including genital, mesenteric, and axillary lymph nodes, spleen, vagina and cervix compared to levels of WT-SIV in plasma. Plasma viral load value listed as 10 were below the limits of detection of the assay. A significant positive association was observed between the plasma viral load and MamuA1/Gag tetramer^+^ cells in lymph nodes.

We compared levels of WT-SIV RNA in plasma (Table 1) to the tissue densities of Mamu-A1/Gag- and Mamu-A1/Tat-tetramer^+^ T cells detected in tissues to determine if there were correlations between the quantities of virus-specific CD8 T cells and plasma WT-SIV virus load in the three animals (56.05, 434.01, and 389.02) in which WT-SIV was detectable. We found a significant positive association between Mamu-A1/Gag binding cells within the lymph nodes and plasma viral loads (genital p = 0.025, mesenteric p = 0.00032, and axillary p = 0.017) (Figure 8); and levels of Mamu-A1/Gag and Mamu-A1/Tat binding cells within individual animals were highly associated (p = 0.0072).

**Table 1:** Copies of WT SIVmac251 RNA per ml plasma.

Animal ID	Days post-challenge WT SIVmac251	Plasma VL
143-09	4 & 5	<30
528-00		<40
338-02	7	<40
56-05		230
434-01	11	580
389-02	14	4.6×10^4^
109-05		<30

<30 and 40 were the limits of detection of the assay.

## Discussion

In this study, we determined by IST staining, the number and location of tetramer^+^ SIV-specific CD8 T cells at the critical early times and sites in the genital and lymphoid tissues where respectively infection begins and to which it is disseminated in unvaccinated animals. We were particularly interested to determine if SIVΔnef vaccination would convert the previously described “too little, too late” CD8 T cell response observed in unvaccinated animals [22], [23] to an “enough and soon enough” response in which virus-specific CD8 T cells would constitute a resident or rapid responder population at the portal of entry to control infection there, or contain infection after virus spread to the lymphoid tissues. The detection reported here of Mamu-A1/Gag and Mamu-A1/Tat tetramer^+^ CD8 T cells distributed amongst other CD8 T cells in lymphoid and genital tissues at all time points examined, including the time of vaginal challenge, stands in stark contrast to our previous observation in unvaccinated vaginally inoculated animals in which SIV-specific CD8 T cells were not detectable in genital or lymphoid tissues until the second week following vaginal inoculation [22], and is consistent with the concept of a resident population of cells operating at an advantage against the small populations of infected cells in the early stages of infection. Our findings are consistent too with our previous studies in animals vaccinated with an attenuated SHIV and vaginally challenged with SIV in which with a resident population of virus-specific CD8 T cells in genital and lymphoid tissues was associated with protection [34].

The numbers and locations of the tetramer^+^ cells, however, cannot by themselves account for the maturation of protection between 5 and 20 weeks post-vaccination, since the decrease between 5 and 20 weeks in the numbers of tetramer^+^ cells, and tetramer^+^Ki67^+^ cells is inversely related to the increasing protection, and is most consistent with the contraction phase of an immune response that parallels the declining levels of SIVΔnef replication in the tissues. Furthermore, the density of the virus-specific CD8 T cell population patrolling the portal of entry in the vaccinated animals is at least five-fold smaller than the density of the populations achieved by three weeks following vaginal inoculation in unvaccinated animals that was associated with 50 to 100-fold reductions in tissue VL from peak levels [24].

Virus-specific CD8 T cells, nonetheless, could be involved in the maturation of protection between 5 and 20 weeks in two ways. First, there could be a change in the “quality” of the CD8 T cells as effectors that correlates at 20 weeks with phenotypic changes in the virus-specific CD8 T cells. Second, there could be a change in non-T cell immune components of the immune system such as the maturation of a vaccine-induced antibody response at the portal of viral entry that might help reduce the establishment and expansion of founder populations of infected cells, thereby creating an environment in which virus-specific CD8 T cells would be operating at numerically superior numbers compared to any infected targets in the early stages of infection at 20 weeks but not at 5 weeks. Paradoxically, in this hypothetical reconstruction of the correlates of the maturation of protection, the marginally significant increase in Tat tetramer^+^ cells at the portal of entry, and positive associations between plasma VL and tetramer^+^ cells in lymphoid tissues could actually represent a failure of first line defenses to successfully fully contain infection. The role of the virus-specific CD8 T cell then becomes the more conventional one of partial control of systemic infection, with the increased levels of tetramer^+^ cells reflecting cells responding to antigenic drive from the breakthrough infection with the challenge virus. It will thus be of interest in future studies to design vaccine strategies that would be more effective than SIVΔnef in inducing and maintaining a larger population of effector CTLs that would work in concert with the local humoral immune response to fully contain and eliminate infected cells at the portal of entry.

## Supporting Information

Figure S1 **Localization of SIV-specific CD8 T cells in ileal Peyer's patches.** The representative images shown are from animal #174.96 stained with Mamu-A1/gag tetramers (red), CD20 antibodies (green), and CD3 antibodies (blue). Tetramer^+^ cells were localized throughout the Peyer's patches of ileum tissue sections with other T cells around as well as within B cell follicles. The left shows a montage of several confocal Z-scan fields stitched together, collected with a 20× objective.
(TIF)
