# Supplementary figures and images for: Location and Dynamics of the Immunodominant CD8 T Cell Response to SIVΔnef Immunization and SIVmac251 Vaginal Challenge
Source: PLoS One. 2013 Dec 9;8(12):e81623. doi: 10.1371/journal.pone.0081623 (PMC3857218; doi:10.1371/journal.pone.0081623)

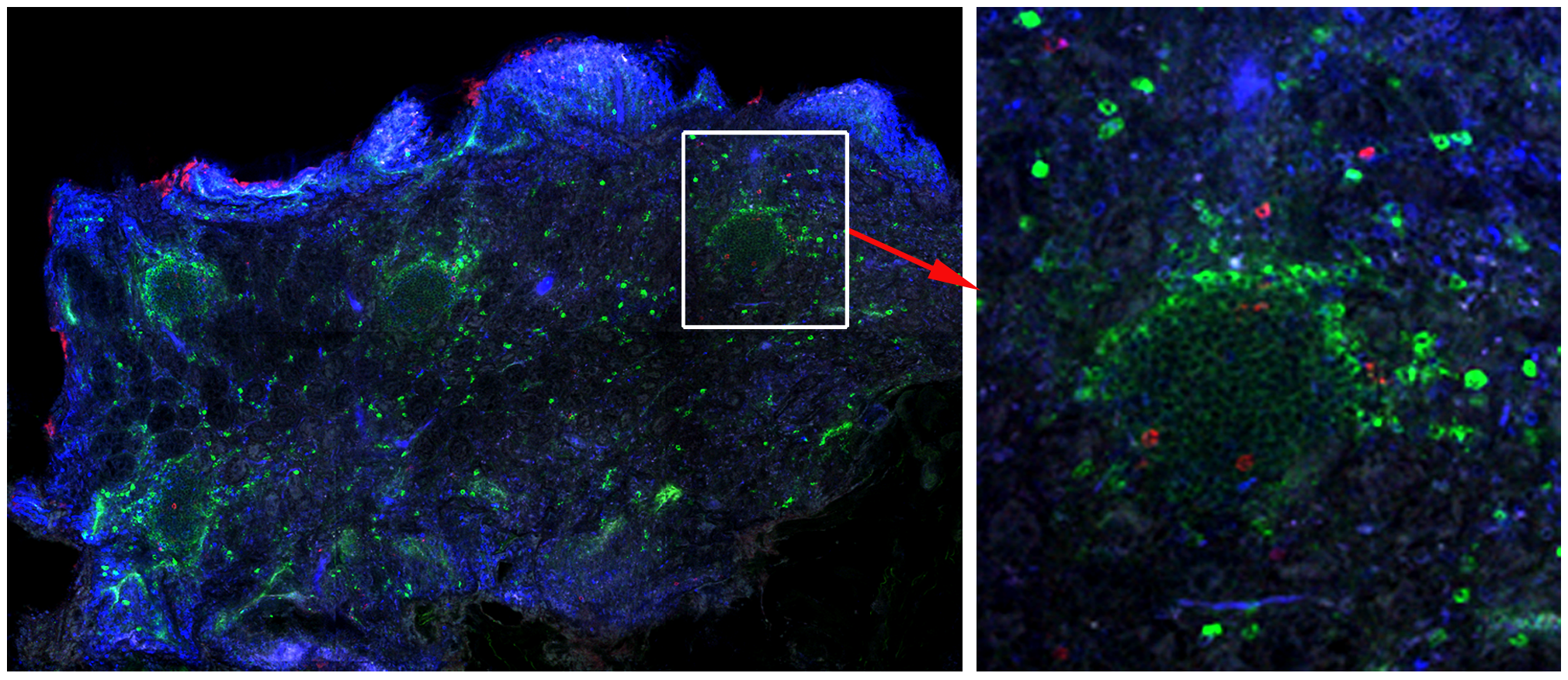

Supplement: Figure S1 — Localization of SIV-specific CD8 T cells in ileal Peyer's patches. The representative images shown are from animal #174.96 stained with Mamu-A1/gag tetramers (red), CD20 antibodies (green), and CD3 antibodies (blue). Tetramer+ cells were localized throughout the Peyer's patches of ileum tissue sections with other T cells around as well as within B cell follicles. The left shows a montage of several confocal Z-scan fields stitched together, collected with a 20× objective. (TIF) [file pone.0081623.s001.tif]
